# COVID-19: instruments for the allocation of mechanical ventilators—a narrative review

**DOI:** 10.1186/s13054-020-03298-3

**Published:** 2020-09-29

**Authors:** Marcelo José dos Santos, Maristela Santini Martins, Fabiana Lopes Pereira Santana, Maria Carolina Silvano Pacheco Corrêa Furtado, Fabiana Cristina Bazana Remédio Miname, Rafael Rodrigo da Silva Pimentel, Ágata Nunes Brito, Patrick Schneider, Edson Silva dos Santos, Luciane Hupalo da Silva

**Affiliations:** 1 Research Group “Bioethics and Administration: Teaching and Health Care”, Nursing School of University of São Paulo, São Paulo, SP Brazil; 2 Departamento de Orientação Profissional, Escola de Enfermagem da Universidade de São Paulo, Rua Dr. Enéas de Carvalho Aguiar, 419, CEP – 05403-000 Cerqueira Cesar, São Paulo, SP Brazil

**Keywords:** Pandemics, Health care rationing, Decision making, Ethics

## Abstract

After the World Health Organization declared COVID-19 to be a pandemic, the elaboration of comprehensive and preventive public policies became important in order to stop the spread of the disease. However, insufficient or ineffective measures may have placed health professionals and services in the position of having to allocate mechanical ventilators. This study aimed to identify instruments, analyze their structures, and present the main criteria used in the screening protocols, in order to help the development of guidelines and policies for the allocation of mechanical ventilators in the COVID-19 pandemic. The instruments have a low level of scientific evidence, and, in general, are structured by various clinical, non-clinical, and tiebreaker criteria that contain ethical aspects. Few instruments included public participation in their construction or validation. We believe that the elaboration of these guidelines cannot be restricted to specialists as this question involves ethical considerations which make the participation of the population necessary. Finally, we propose seventeen elements that can support the construction of screening protocols in the COVID-19 pandemic.

## Introduction

SARS-CoV-2 is a highly transmissible virus that causes COVID-19, a disease that can evolve to a severe clinical state due to a chronic systemic inflammatory response which can lead to acute respiratory distress syndrome [1–3].

After the World Health Organization declared COVID-19 to be a pandemic [4], new public health policies aimed at protecting the population became essential. However, the adoption of ineffective public policies may have contributed to the increase in the number of cases and the consequent overload of the health system [5, 6].

Studies show that 17 to 35% of patients affected by this disease require hospitalization in intensive care units [7, 8] and that from 9 to 19% need invasive mechanical ventilation (IMV) [7, 9], during a period which can vary from 2 to 4 weeks [10]. Estimates indicate that countries such as the USA [11, 12] and Brazil [13] may not have enough invasive mechanical ventilators to attend all of the patients who need this resource for COVID-19 treatment. This was the situation in Italy, where doctors had to decide which patient would receive IMV [14]. Decision making in the allocation of scarce resources in pandemics, in addition to directly impacting people’s lives, can lead the professional to a situation of moral suffering [15].

Given this scenario, it is urgent to build managerial and ethical strategies in order to ration scarce resources [16]. Thus, this study aims to identify instruments, analyze their structures, and present the main criteria used in the screening protocols in order to help the development of guidelines and policies for the allocation of mechanical ventilators in the face of the COVID-19 pandemic.

## Methods

The synthesis of evidence was performed based on articles that presented instruments for decision making in the allocation of mechanical ventilators, in contexts of respiratory pandemics. The databases searched were ASSIA, Embase, PubMed, Scopus, and academic platform ScienceDirect. An additional file shows the exact search strategies (see Additional file 1). Only articles with the context of respiratory pandemics, primary studies, theoretical, opinion, and consensus were included, regardless of language and date of publication. We screened the results on the title and abstract for relevant information. The selected articles were read in full. From the articles and reviews found in this search, we used the snowball strategy, checking useful references and similar articles and retrieving those that were considered relevant. Guidelines from international organizations were used to identify more articles. This search was last updated on April 15, 2020.

### Instruments

The development of instruments to allocate scarce resources increases in periods of pandemics, as occurred with influenza [ 17–24], with severe respiratory distress syndrome (SARS) [18, 23] and nowadays with COVID-19 [25–27].

After the SARS outbreak in 2003 [28], a pioneering guideline for triage in intensive care in pandemics [29] was developed and published, and this became a reference for the development of new instruments [17–27, 30–32]. An additional file shows a summary of each instrument identified in this investigation in more detail (see Additional file 2).

Any guidelines take into account the expected prognosis. Thus, data on the prognosis of patients with COVID-19 is needed to update and improve existing protocols. However, these data may be unavailable due to the short period since the onset of the disease [33].

Some of the instruments found in this study [17, 20–27, 29–31], direct screening for access to intensive care (intensive care beds, hemodynamic and respiratory support, antibiotic therapy, renal replacement therapy), while others [18, 19, 32] deal specifically with the allocation of invasive mechanical ventilators.

The construction of these instruments was based on expert opinions and consensus. To guarantee quality and rigor in this process, Ornelas et al. [34] systematized a method composed of the domains: methodology, usability, validity, bias, and summative. In order to assess the instruments included in this review, a score was established, based on the usability domain [34], for each instrument presented (Table 1).

**Table 1:** Presentation of instruments for decision-making

Authors	Country/year	Community participation	Clinical Instrument	Patient	Reassessment	Stratification Criteria	Tiebreakers	Ventilation withdrawal	U^**c**^
Ardagh [17]	NZ/2006	No	Uninformed	Adult/pediatric	Uninformed	Subjective	Yes	No	6/8
Christian et al. [29]	CA, US/2006	No	SOFA^a^, NYHA Classification; Child-Pugh	Adult	48 h^b^ e 120 h	Objectives	No	Yes	8/9
Hick & O’Laughlin [18]	US/2006	No	SOFA	Adult/pediatric	Uninformed	Objectives	No	Yes	9/9
Devereaux et al. [30]	US/2008	No	SOFA^a^	Adult/pediatric	Daily	Objectives	Yes	Yes	9/9
Powell, Christ, Birkhead [19]	US/2008	Yes	SOFA, MELD, NYHA Classification	Adult	48 h e 120 h	Objectives	No	Yes	8/9
Lin & Anderson-Shaw [20]	US/2009	No	SOFA^a^; MELD; NYHA Classification	Adult	48 h e 120 h	Objectives	No	Yes	8/9
Frolic, Kata, Kraus [21]	CA/2009	No	SOFA; ISS; NYHA Classification; Child-Pugh	Adult	48 h e 120 h	Objectives	Yes	Yes	9/9
White et al. [22]	US/2009	No	SOFA	Adult	Uninformed	Objectives	Yes	Yes	8/9
Christian et al. [23]	CA, CN, US, UK,IL/2010	No	SOFA^a^, NYHA Classification; Child-Pugh; TRISS	Adult	48 h e 120 h	Objectives	No	Yes	8/9
Christian et al. [31]	CA, IL, US, CN/2014	No	SOFA, MELD; NYHA Classification	Adult/pediatric	72 h and 96 h or as needed	Objectives	Yes	Yes	9/9
Gall et al. [24]	US/2016	No	POD; DV	Pediatric	Uninformed	Objectives	No	No	6/9
Daugherty Biddison et al. [32]	US/2019	Yes	SOFA; PELOD-2; NYHA Classification; Child-Pugh	Adult/pediatric	24 h, 48 h e 120 h	Objectives	Yes	Yes	9/9
Rubio et al. [25]	ES/2020	No	SOFA, Charlson Comorbidity Index (CCI), NECPAL	Adult/pediatric	Daily	Objectives	No	Yes	8/9
White & Lo [26]	US/2020	No	SOFA; LAPS2; MELD; NYHA Classification; ECI; COPS2	Adult/pediatric (from 12 years)	Daily	Objectives	Yes	Yes	9/9
Swiss Academy Of Medical Sciences [27]	CH/2020	No	NYHA Classification; Child-Pugh; KDIGO	Adult	48 h	Objectives	No	Yes	5/9

Note: Country: *NZ* New Zealand, *CH* Switzerland, *ES* Spain, *CN* China, UK United Kingdom, *IL* Israel, *CA* Canada, *US* United State of America

Clinical instrument: *SOFA* Sequential Organ Failure Assessment, *PELOD2* Pediatric Logistic Organ Dysfunction 2, *LAPS2* Laboratory-Based Acute Physiology Score, *MELD* Model for End-Stage Liver Disease, *NECPAL* Palliative Needs, *NYHA Classification* New York Heart Association Classification, *ISS* Injury Severity Score, *TRISS* Trauma and Injury Severity Score, *KDIGO* Kidney Disease Improving Global Outcomes, *ECI* Elixhauser Comorbidity Index, *COPS2* Comorbidity Point Score, *POD* Probability of Death, *DV* Days on Mechanical Ventilation (DV)

SOFA^a^ = Adapted SOFA; h^b^ = hours; **U**^**c**^ **=** Usability

In general, the structure of the instruments includes clinical, non-clinical, and tiebreaker criteria, which are permeated by ethical aspects for decision making in the allocation of scarce resources [17–27, 29–32] (Table 2).

**Table 2:** Examples of the application of criteria found in the screening instruments in the allocation of mechanical ventilators

Section	Ethical values	Criteria found in the instruments	History	Commented examples
**Clinical criteria**	“Save more lives”	Instruments with exclusion [18–21, 23, 25, 27, 29–32]	**Patient A:** 26 years old, affected by severe trauma, with respiratory failure. **Patient B:** 65 years old, without comorbidities, diagnosed with COVID-19 and with respiratory failure.	The resource would be allocated to patient B as a result of patient A’s poor prognosis and the high probability of death. Severe trauma is an exclusion criterion in most instruments.
	“Save more lives”	Instruments without exclusion (multiple principles) [22, 26]	**Patient A:** 49 years old with mild COPD, benzodiazepine intoxication and respiratory failure, SOFA score: 4. **Patient B:** 61 years old, with severe Alzheimer’s disease, chronic renal failure and on dialysis, and diagnosis of COVID-19 with respiratory failure, SOFA score: 10. **Patient C:** 64 years old, without comorbidities, with 60% of the body area injured by 2nd and 3rd degree burns, with sepsis, multiple organ failure and respiratory failure, SOFA score: 12: 12.	The resource would be given to Patient A, to the detriment of Patients B and C, who had lower prioritization due to the short and long term prognosis and life cycle. Despite health conditions and severe comorbidities, all patients are included in the screening protocol.
**Non-clinical criteria**	“Equity”	Life cycle [21]	**Patient A:** 75 years old. No comorbidities, diagnosis of COVID-19 with respiratory failure. SOFA: 6. **Patient B:** 45 years old. No comorbidities, diagnosed with COVID-19 and respiratory failure, SOFA: 6.	Patient B would have a higher priority to receive the resource, based on the logic that everyone should have the same opportunity to live all the cycles of life.
**Tiebreaker criteria**	“Equality”	Ballot [17, 21, 22, 26, 31, 32]	**Patient A:** 20 years old, without severe comorbidities, diagnosed with COVID-19 with respiratory failure, SOFA score 7. **Patient B:** 39 years old, diagnosed with COVID-19 with respiratory failure, without severe comorbidities. SOFA 7.	Patients had the same priority score, both in terms of prognosis and life cycle (0–49 years). The resource would be allocated based on chance, in a fair and transparent way.

Note: *SOFA* Sequential Organ Failure Assessment, *COPD* chronic obstructive pulmonary disease

### Clinical criteria

The clinical evaluation criteria for screening patients in situations of scarcity of resources aim to identify the patients most likely to survive hospital discharge [35]. The prognosis is one of the parameters used to support decision making in the allocation of intensive care in order to balance benefits and harm, in addition to determining when treatment becomes futile [36]. However, the prognostic assessment carried out by different members of the same multidisciplinary team may reach different conclusions [37, 38]. It is, therefore, an imprecise parameter [39].

In order to increase the accuracy and safety of the prognosis, disease severity scores, and clinical forecasting models have been developed, but so far, they have not been completely satisfactory [40]. It is important to emphasize that in order to determine the prognosis, it is not recommended to consider only the probability of survival, since measures of functional results and quality of life are also aspects considered essential for both patients and society [41].

In this review, the instruments which were found used, in order to determine the probability of survival, the Sequential Organ Failure Assessment score (SOFA) [18–23, 25, 26, 29–32], the Laboratory Acute Physiology Score (LAPS2) [26], the calculation for the prediction of the Mortality Risk Index associated with the number of days on mechanical ventilation [24], and/or the Pediatric Logistic Organ Dysfunction 2 (PELOD-2) [32].

SOFA was the most frequently used instrument for decision making in the allocation of critical care [18–23, 25, 26, 29–32]. After the experiences with the H1N1 pandemic, the use of this tool as a screening strategy gained popularity, which is attributed to the objectivity of the instrument, its low cost, and its use of complementary exams which are not complex [18–23, 25, 26, 29–32].

However, the use of SOFA to screen COVID-19 patients has been questioned [33] and not recommended [42]. Some studies [43–45] show that, due to the cutoff points adopted in some of the protocols, SOFA may not be useful for screening decisions in the COVID-19 pandemic [33].

One study [33] suggests that these instruments should not use a single scoring tool but rather a combination of scoring systems and mortality predictors in intensive care, such as disease-specific indicators for non-COVID-19 conditions, frailty score, comorbidity rates, and clinical judgment [33].

PELOD-2 was developed and validated to assess the severity of pediatric multiple organ dysfunction syndrome cases [46], based on the PELOD created in 1999 [47] and validated in pandemic settings [48]. The PELOD-2 score, different from its original version, includes mean arterial pressure and lactatemia in cardiovascular dysfunction and does not include liver dysfunction [46]. Among its limitations, the need for laboratory tests for evaluation has been highlighted [32, 49]. In this area, the Pediatric Emergency Task Force for Critical Mass Care developed specific recommendations for the organization, screening, and treatment in scenarios that require the management of scarce resources [50].

It is noteworthy that, currently, it is unclear whether any severity score for pediatric or neonatal disease provides valid prognostic measures for COVID-19 patients [51].

The screening structure can combine several evaluation parameters specific for diseases or health conditions, which generally integrate the inclusion and exclusion criteria. This screening should be based on clear and accurate evidence to avoid arbitrary admissions and prevent the prolonged treatment of patients whose survival is unlikely [52].

The inclusion criteria must be objective [18–27, 29–32] and facilitate the suitable and rapid referral of potential survivors during pandemics or major disasters [53]. Exclusion criteria should identify patients who are unlikely to obtain benefits through intensive care [53], as recommended by the European Society of Intensive Care Medicine task force [23, 54].

In general, studies indicate the following inclusion criteria: respiratory failure [18–21, 23, 25, 27, 29–31] and hypotension [21, 23, 27, 29–31].

The most common exclusion criteria were pulmonary impairment [41], trauma (Injury Severity Score [21]; Trauma and Injury Severity Score [23]), renal dysfunction (Kidney Disease Improving Global Outcomes [27]), hepatic (Model for End-Stage Liver Disease [19, 20, 26, 31]; Child-Pugh classification [21, 23, 27, 29, 32]) and cardiac classification (New York Heart Association classification [19–21, 23, 26, 27, 29, 31, 32]), and comorbidities (Charlson comorbidity index [25]; Elixhauser Comorbidity Index [26]; Comorbidity Point Score 2 [26]). In addition, there are studies [22, 26] that use some of these criteria in order to prioritize patients.

In order to mitigate prolonged treatments and maximize the chance of benefit, it is recommended that patients should be continuously reassessed as this allows for the confirmation of clinical evolution and opens the possibility of the redistribution of the mechanical ventilator to patients who would have a better survival prognosis [35]. Some studies mention that this reassessment must be carried out daily [25, 26, 30], and others indicate different times: intervals of 24, 48, and 120 h [32]; 48 and 120 h [19–21, 23, 29]; and 72 and 96 h [31].

### Non-clinical criteria

In COVID-19 patients, the advanced age of the patients suggests a higher mortality rate [14, 43, 55]. This factor seems to be important in the utilitarian approach but should not be the only criterion to determine screening decisions [56, 57].

The use of age [21, 25, 27, 29, 30, 32] as a non-clinical criterion has been strongly contested for violating the notions of equality and justice [58, 59]. Even so, there are authors who defend the use of age only as a tiebreaker criterion [59] and others who refute even that use, as they consider that a criterion is seen as discriminatory and cannot suddenly become ethically appropriate [60].

This criterion seems to be associated with the idea of frailty [61] and chronic comorbidities [62], suggesting that increasing age leads to a higher probability of death [47]. Replacing the age criterion with the frailty assessment seems to better determine the risk of worse health outcomes and avoids age discrimination in the screening protocols [63]. On the other hand, carrying out assessments based on frailty to determine the probability of survival may constitute indirect discrimination as the elderly and people with specific disabilities may be considered more fragile [64].

The use of the life cycle [21] maintains that everyone should have the same opportunity to live all the cycles of life. Resources must then be distributed to ensure that those who have not yet lived this life are given priority to those who have already managed to do so [65]. The fair inning argument puts young people at an advantage over old people in the context of healthcare decision-making—a view that generally receives implicit public approval based on an efficiency perspective since younger people are more able to gain years of their life, which makes any other choice difficult to justify in social and ethical terms [66].

Pregnancy [32] is a unique state in which clinical decisions directly affect the state of health and the expectation of survival not only for the mother, but also for the fetus inside her [32]. These women represent a particular population, which historically has been disproportionately affected in seasonal outbreaks and in the influenza pandemic [67, 68]. A recent forecast suggests that COVID-19 will affect millions of women in the USA in the hospital when they are giving birth [69]. One study states that prioritization should be based on the criterion of viable pregnancy [67] and another on pregnancy as long as the fetus is healthy [32]. This study also highlights that there is a possibility that pregnant women with comorbidities are given a lower priority [67].

The net benefit [17] criterion assesses the difference between the estimated benefit of accessing the resource and the harm caused by not accessing it [17]. The instrumental value [21] considers professionals, among whom are health professionals, vaccine researchers, public health professionals, and others essential to deal with a disaster scenario [21, 27]. This effect is supported by the utilitarian principle of triage as it prioritizes access to the resources for people with essential skills and knowledge to save others’ lives (assuming the recovery of the multiplier to exercise their knowledge), thus multiplying the net benefit to society [21, 70].

Having someone dependent on care [21] is considered a criterion in the triage model as it is considered that this criterion should be prioritized due to the fact that less harm will be caused to families and society [21]. The existence of ADLW [27] should be verified as people are more aware of the importance of preparing this document, as well as having conversations with their families about the adoption of measures at the end of life [71]. The option of not using IMV, painful or exhausting treatment, or even the wish to not resuscitate in case of cardiac arrest shortens the triage process, facilitates decision making, and directs the patient’s referral to palliative care.

It is important to emphasize that the screening instruments must take into account human rights in terms of the set of protections and rights offered to all people [59]. The fundamental principles of dignity, non-discrimination, equal opportunities, and accessibility should be major factors when planning the allocation of scarce resources [72]. In this sense, ventilators should not be allocated on the basis of morally irrelevant aspects such as sex, race, religion, financial condition, social relations, citizenship, and physical or intellectual disabilities [26, 73]. It should be emphasized, therefore, that these people should not suffer any type of discrimination due to their condition and should be included in the screening protocols [59].

### Tiebreaker criteria

After applying the specific decision-making criteria, there may be a tie in prioritizing the resource. In order to solve this problem, the instruments suggest that the tiebreaker takes into account the following criteria: life cycle [26, 32], instrumental value [26], gross prioritization score [17, 30–32], order of arrival, and ballot [17, 21, 22, 26, 31, 32].

In some studies, the life cycle [26, 32] and instrumental value [26] were considered as tiebreaker criteria; however, they can also be used, as previously mentioned, in the application of criteria which are supplementary to clinical criteria.

Instrumental value [26] does not judge people by their value but rather by their utilitarian basis [74]. The application of this criterion in the tiebreaker encounters obstacles when the people evaluated have the same utilitarian value.

The criterion of the gross prioritization score [26] was listed as a tiebreaker criterion in a study that uses a system of multiple principles, in which it considers the lowest gross value of this score in the tiebreaker.

Order of arrival, “first come, first served” is mentioned as a tiebreaker criterion by some authors [17, 30–32]. However, there are studies that state that this criterion is not fair as those with greater social resources will have quicker access than those who have little access to health care [75–77].

The ballot [17, 21, 22, 26, 31, 32] is a tiebreaker criterion considered transparent and impartial in random selection [21] and also seen as a fair way to ensure that all patients have equal access to life-saving care [74]. This criterion requires little knowledge of the recipients, can be applied quickly, and resists corruption. On the other hand, ballots—and the egalitarian principles of justice in general—are insensitive to factors that are also intuitively important to many, such as the needs of patients and the likelihood of obtaining benefits from the treatment [75, 76].

The tiebreaker criteria identified in the analyzed instruments are varied, have strong points, disadvantages, and show a lack of consensus.

### Ethical aspects

In health, one of the ethical dilemmas that emerges is who to give priority to in order to receive assistance/resources [14, 41]. In general, public health measures often adopt a utilitarian orientation that has social utility as a fundamental principle. This principle states that actions are ethically correct when they tend to promote the greatest amount of pleasure (happiness, well-being) of all those whose interests are at stake [78].

Life is a social value [79]. Thus, in the face of a catastrophic situation such as that of a pandemic, the main aim of a society will be to make every effort to use all its resources not only to “save lives,” but to save “the greatest possible number of lives.” This scenario becomes complex in situations of the scarcity of resources because criteria, and choices will inevitably have to be made about who will and who will not have access to them. In this sense, distributive justice, which guides the utilitarian paradigm of “the greatest good for the greatest number of people” emerges as a principle for decision making, as evidenced in the guidelines specified in several studies [17–23, 25–27, 29, 31, 32].

A number of studies [18–23, 26, 27, 29, 31, 32] based on this paradigm developed instruments with clinical criteria based on the probability of the survival of people who need the resource and/or the identification of comorbidities, considering that those in better health will have greater chances of life and a possible rapid response to the use of the resource, thus freeing it to be used for other people. However, it is important that the screening instruments merge clinical and ethical criteria to create a balanced evaluation process [22].

Although the application of criteria based on the utilitarian approach is more frequent, it is also possible to identify in the instruments [17, 21, 22, 26, 31, 32] criteria based on the egalitarian approach that argues that all people have incomparable value. From this perspective, no one has more or less value than anyone else, and neither does this value increase or decrease based on their quality of life related to health, personal satisfaction or well-being, intelligence, talent, or instrumental value [80]. Egalitarianism is based only on the consideration of need and rejects the consideration of the probability of survival, longevity, or quality of life typical of the utilitarian approach [64].

The utilitarian and egalitarian approaches can both be seen as inconsistent and unjustified [64], and therefore, seeking a balance between equality and utility may be the way to build a new ethical structure to allocate scarce resources [64].

In general, a screening system is built in order to meet the values of human life, health, an efficient use of resources, and justice [79]. Even so, it is possible to find triage instruments in the allocation of different scarce resources that present discriminatory criteria [81]. From an ethical point of view, the exclusion of patients with comorbidities that do not influence their probability of survival is questionable and should not be considered [82].

Some of the studies analyzed considered or associated with clinical criteria, values based on the principle of equity—specifically based on the life cycle, explained by the guideline “maximize the chances of individuals to live each stage of life” [22, 25]; those based on the principle of reciprocity that values those who demonstrate altruism taking care of others at the expense of their own safety during a pandemic [17, 21]; those based on the principle of proportionality, that is, those with a better prognosis will have easier access to resources [25]; and those based on the principle of autonomy [83]—aimed at those who expressed their choice in the ADLW [27].

It is important to consider that the existence of instruments built from literature reviews and expert consensus, but without popular participation to validate the guidelines, may not represent the real values of the society in question. There is no doubt that the public may have a limited understanding and, consequently, less possibility of contributing in terms of the pertinence and application of the clinical criteria of the specialized medical domain, which can be seen in the instruments; however, society can greatly collaborate in the discussion, reflection, and definition of “non-clinical” criteria as these clearly involve “social values.”

Few instruments have been developed with the participation of the public [19, 32]. This fact attracts attention since it is only through the collective construction of these guidelines and public policies that it is possible to minimize the paternalistic bias present in the construction of the instruments developed for this purpose. Society can take part directly or through elected representatives, and public opinion surveys can also guide the construction of instruments aimed at allocating scarce resources [84].

Transparency and social inclusion are essential conditions in the process of building any ethical structure for decision making in situations of scarce resources because it is of paramount importance that citizens trust health institutions and the provision of care in the system of which they are part [85].

Some of the instruments [18–23, 25–27, 29–32] evaluated addressed the notion of removing invasive mechanical ventilators from patients initially screened with a higher probability of mortality in order to benefit patients with a high probability of survival. This proposal aims at the optimization of resources, and although it can ethically be defended from the utilitarian point of view, it places the health professional in a disturbing situation and is therefore difficult to implement. It should be noted that these instruments rarely provide clinical or ethical guidance on how these decisions should be applied in practice, discussed with family members, and whether they meet legal standards [86–89].

It is positive that some guidelines have highlighted the duty to care for those who do not meet the criteria for using scarce resources through actions based on comfort and pain relief (palliative care) [17, 19, 20, 23, 29, 30]. On the other hand, there is a considerable lack of clarity regarding the values and ethical principles on which these instruments are based as they are presented disconnectedly in the body of the articles and are often not linked to the criteria and actions established in the guidelines.

It should be emphasized that an ethical problem always presents a conflict of values, duties, or principles, and therefore, in situations of ethical conflict, decisions cannot be said to be “right” or “wrong.” What is necessary is that they are all reasonable and that the reasons in their favor can be understood so the decisions taken must be prudent [90].

### Implications for practice

The instruments identified for allocating invasive mechanical ventilators have different structures and criteria, which make their reproducibility limited as the local context must be considered. In order to help the construction of screening protocols for the allocation of invasive mechanical ventilators, we have summarized the review and suggested a number of items to consider (Table 3).

**Table 3:** Items to be considered in the construction of the screening protocol

• Make an extensive review of the literature.	
• Examine the existing ethical structures.	
• Provide a basis for the chosen ethical structures.	
• Define inclusion criteria.	
• Establish complementary or non-clinical criteria.	
• Combine point systems and mortality predictors, which should be validated, clear, and objective, in intensive therapy.	
• Define a prioritization score	
• Establish reevaluation periods.	
• Use a multiple principle prioritization structure.	
• Enable the participation of stakeholders (specialists, clinicians, decision makers, and society) in the definition of the ethical structure and clinical, non-clinical, and tiebreaker criteria.	
• Establish tiebreak criteria.	
• Define alternative treatments/options for those who will not benefit from the resource.	
• Develop specific guidelines for children.	
• Pay attention to the usability of the screening instrument.	
• Develop an algorithm for the screening instrument.	
• Make the established screening policy and criteria public.	
• Periodically update the protocol.	

## Conclusion

Decision making in allocating ventilators is a complex practice, especially in pandemic settings. For this purpose, there are instruments with varying criteria, which address this difficult and controversial topic.

A number of these instruments have poor scientific evidence and until now have not included the participation of the public or have not yet been submitted to validation by society, which is the affected population and is thus interested in the context. Therefore, certain established criteria may not represent social values and may even be discriminatory.

We believe that it is important to prepare comprehensive public policies for all health services in advance and consider preventive actions to halt the spread of the disease in pandemic scenarios, as well as measures to allocate scarce resources that include clear, objective, and transparent screening instruments which are easy to use in order to avoid dubious interpretations and mistakes regarding the application of the established criteria. Finally, the elaboration of these guidelines cannot be restricted to specialists as this question involves ethical considerations which make the participation of the population necessary.

## Supplementary information

**Additional file 1.** Search terms and strategies.

**Additional file 2.** Summaries of the instruments presented in the article.

## Data Availability

All data analyzed during this study are included in this published article [and a supplementary information file].

## Abbreviations

ADLW: Advance directives and living will
LAPS2: Laboratory acute physiology
PELOD-2: Pediatric logistic organ dysfunction 2
SARS: Severe respiratory distress syndrome
SOFA: Sequential Organ Failure Assessment
