# Supplementary material for: COVID-19: instruments for the allocation of mechanical ventilators—a narrative review
Source: Crit Care. 2020 Sep 29;24:582. doi: 10.1186/s13054-020-03298-3 (PMC7522926; doi:10.1186/s13054-020-03298-3)
Supplement: Supplementary file 1 — Additional file 1. Search terms and strategies. [file 13054_2020_3298_MOESM1_ESM.docx]

**SUPPLEMENTARY APPENDIX 1**

**Search terms and strategies**

The Medical Subject Headings (MeSH) search terms used were "Pandemics" [MeSH]; "Coronavirus" [MeSH]; "Severe Acute Respiratory Syndrome" [MeSH]; "Decision making" [MeSH]; "Resource Allocation" [MeSH]; "Ventilators, Mechanical" [MeSH]; "Ethics" [MeSH]; "Bioethics" [MeSH]. Non-MeSH search terms used were: epidemic, COVID-19 [Supplementary Concept], 2019-nCoV, SARS-CoV-2, coronavirus infection, severe acute respiratory syndrome coronavirus 2 [Supplementary Concept], Wuhan, artificial ventilation, mechanical ventilation, mechanical ventilators. The terms were combined using the boolean operators "AND" and "OR".

**ASSIA**

((Epidemics OR Pandemics) AND "Severe Acute Respiratory Syndrome" AND (Ethics OR bioethics)) OR ((Coronavirus OR covid-19 OR 2019-ncov OR sars-cov-2) AND (Ethics OR bioethics))

**Embase**

('coronavirus infection'/exp OR 'covid 19' OR 'sars cov 2' OR '2019ncov') AND 'ethics'/exp AND 'resource allocation'/exp OR ('ethics'/exp AND 'resource allocation'/exp AND 'severe acute respiratory syndrome'/exp) OR (('epidemic'/exp OR 'pandemic'/exp) AND 'resource allocation'/exp AND ('artificial ventilation'/exp OR 'mechanical ventilation')) OR ('epidemic'/exp AND 'decision making'/exp AND ('artificial ventilation'/exp OR 'mechanical ventilation')) OR ('epidemic'/exp AND 'resource allocation'/exp AND 'severe acute respiratory syndrome'/exp)

**Pubmed**

((((((("Epidemics"[Mesh] OR "Epidemics"[tw]) AND ("Ventilators, Mechanical"[Mesh] OR "mechanical ventilators"[tw])) AND (("Ethics"[Mesh] OR "ethics"[tw]) OR ("Bioethics"[Mesh] OR "Bioethics"[tw]))) OR ((("Epidemics"[Mesh] OR "Epidemics"[tw]) AND ("Severe Acute Respiratory Syndrome"[Mesh] OR "Severe Acute Respiratory Syndrome"[tw])) AND (("Ethics"[Mesh] OR "ethics"[tw]) OR ("Bioethics"[Mesh] OR "Bioethics"[tw])))) OR ((("Coronavirus"[Mesh] OR "Coronavirus"[tw] OR ("COVID-19"[All Fields] OR "COVID-2019"[All Fields] OR "severe acute respiratory syndrome coronavirus 2"[Supplementary Concept] OR "severe acute respiratory syndrome coronavirus 2"[All Fields] OR "2019-nCoV"[All Fields] OR "SARS-CoV-2"[All Fields] OR "2019nCoV"[All Fields] OR (("Wuhan"[All Fields] AND ("coronavirus"[MeSH Terms] OR "coronavirus"[All Fields])) AND (2019/12[PDAT] OR 2020[PDAT]))) OR ("severe acute respiratory syndrome coronavirus 2"[Supplementary Concept] OR "severe acute respiratory syndrome coronavirus 2"[All Fields] OR "sars cov 2"[All Fields])) AND (("Ethics"[Mesh] OR "ethics"[tw]) OR ("Bioethics"[Mesh] OR "Bioethics"[tw]))) AND ("Resource Allocation"[Mesh] OR "Resource Allocation"[tw]))) OR ((("Coronavirus"[Mesh] OR "Coronavirus"[tw] OR ("COVID-19"[All Fields] OR "COVID-2019"[All Fields] OR "severe acute respiratory syndrome coronavirus 2"[Supplementary Concept] OR "severe acute respiratory syndrome coronavirus 2"[All Fields] OR "2019-nCoV"[All Fields] OR "SARS-CoV-2"[All Fields] OR "2019nCoV"[All Fields] OR (("Wuhan"[All Fields] AND ("coronavirus"[MeSH Terms] OR "coronavirus"[All Fields])) AND (2019/12[PDAT] OR 2020[PDAT]))) OR ("severe acute respiratory syndrome coronavirus 2"[Supplementary Concept] OR "severe acute respiratory syndrome coronavirus 2"[All Fields] OR "sars cov 2"[All Fields])) AND ("Decision Making"[Mesh] OR "Decision Making"[tw])) AND (("Ethics"[Mesh] OR "ethics"[tw]) OR ("Bioethics"[Mesh] OR "Bioethics"[tw])))) OR (("Coronavirus"[Mesh] OR "Coronavirus"[tw] OR ("COVID-19"[All Fields] OR "COVID-2019"[All Fields] OR "severe acute respiratory syndrome coronavirus 2"[Supplementary Concept] OR "severe acute respiratory syndrome coronavirus 2"[All Fields] OR "2019-nCoV"[All Fields] OR "SARS-CoV-2"[All Fields] OR "2019nCoV"[All Fields] OR (("Wuhan"[All Fields] AND ("coronavirus"[MeSH Terms] OR "coronavirus"[All Fields])) AND (2019/12[PDAT] OR 2020[PDAT]))) OR ("severe acute respiratory syndrome coronavirus 2"[Supplementary Concept] OR "severe acute respiratory syndrome coronavirus 2"[All Fields] OR "sars cov 2"[All Fields])) AND (("Ethics"[Mesh] OR "ethics"[tw]) OR ("Bioethics"[Mesh] OR "Bioethics"[tw])))) OR (((("Epidemics"[Mesh] OR "Epidemics"[tw]) OR ("Pandemics"[Mesh] OR "Pandemics"[tw])) AND ("Resource Allocation"[Mesh] OR "Resource Allocation"[tw])) AND (("Ethics"[Mesh] OR "ethics"[tw]) OR ("Bioethics"[Mesh] OR "Bioethics"[tw])))

**Scopus**

( ( TITLE-ABS-KEY ( covid-19  OR  sars-cov2  OR  coronavirus  OR  "2019nCoV" ) )  AND  ( TITLE-ABS-KEY ( ethics  OR  bioethics ) ) )  OR  ( ( ( ( TITLE-ABSKEY ( epidemics  OR  pandemics ) )  AND  ( TITLE-ABS-KEY ( ethics  OR  bioethics ) ) )  AND  ( TITLE-ABS-KEY ( "Severe Acute Respiratory Syndrome" ) ) )  AND  ( TITLE-ABS-KEY ( "resource allocation" ) ) )  OR  ( ( ( TITLE-ABS-KEY ( ethics  OR  bioethics ) )  AND  ( TITLE-ABS-KEY ( "Severe Acute Respiratory Syndrome" ) )  AND  ( TITLE-ABS-KEY ( "resource allocation" ) ) )  OR  ( ( TITLE-ABS-KEY ( covid-19  OR  sars-cov-2  OR  coronavirus  OR  "2019nCoV" ) )  AND  ( TITLE-ABS-KEY ( "resource allocation" ) ) ) )

**ScienceDirect**

(("mechanical ventilators" OR SARS OR "Severe Acute Respiratory Syndrome") AND (ethics OR bioethics) AND ("resource allocation"))(Coronavirus OR covid-19 OR 2019-ncov OR sars-cov-2) AND "resource allocation"(epidemics OR pandemics) AND "mechanical ventilation" AND (ethics OR bioethics) AND ("resource allocation") Title, abstract, keywords: (covid-19 OR sars-cov2 OR coronavirus OR 2019nCoV) AND (ethics OR bioethics)
