# Supplementary material for: COVID-19: instruments for the allocation of mechanical ventilators—a narrative review
Source: Crit Care. 2020 Sep 29;24:582. doi: 10.1186/s13054-020-03298-3 (PMC7522926; doi:10.1186/s13054-020-03298-3)
Supplement: Supplementary file 2 — Additional file 2. Summaries of the instruments presented in the article. [file 13054_2020_3298_MOESM2_ESM.docx]

# SUPPLEMENTARY APPENDIX 2

**Table of contents:**

[**Flowchart 1.** Summary of the instrument presented in the article: Criteria for prioritizing access to healthcare resources in New Zealand during an influenza pandemic or at other times of overwhelming demand^1^ 2](#_TOC_250013)

[**Flowchart 2**. Summary of the instrument presented in the article: Development of a triage protocol for critical care during an influenza pandemic^2^ 4](#_TOC_250012)

[**Flowchart 3.** Summary of the instrument presented in the article: Concept of Operations for Triage of Mechanical Ventilation in an Epidemic^3^ 5](#_TOC_250011)

[**Flowchart 4.** Summary of the instrument presented in the article: Definitive Care for the Critically Ill During a Disaster: A Framework for Allocation of Scarce Resources in Mass Critical Care^4^ 6](#_TOC_250010)

[**Flowchart 5.** Summary of the instrument presented in the article: Allocation of Ventilators in a Public Health Disaster^5^ 7](#_TOC_250009)

[**Flowchart 6.** Summary of the instrument presented in the article: Rationing of resources: ethical issues in disasters and epidemic situations^6^ 8](#_TOC_250008)

[**Flowchart 7.** Summary of the instrument presented in the article: Development of a Critical Care Triage Protocol for Pandemic Influenza: Integrating Ethics, Evidence and Effectiveness^7^ 9](#_TOC_250007)

[**Flowchart 8.** Summary of the instrument presented in the article: Who Should Receive Life Support During a Public Health Emergency? Using Ethical Principles to Improve Allocation Decisions^8^ 10](#_TOC_250006)

**Flowchart 9.** Summary of the instrument presented in the article: Chapter 7. Critical care triage. Recommendations and standard operating procedures for intensive care unit and hospital preparations for an influenza epidemic or mass disaster

^9^........................................................................................................................................11

[**Flowchart 10.** Summary of the instrument presented in the article: Triage: care of the critically ill and injured during pandemics and disasters: CHEST consensus statement^10^ 12](#_TOC_250005)

[**Flowchart 11.** Summary of the instrument presented in the article: Pediatric Triage in a Severe Pandemic: Maximizing Survival by Establishing Triage Thresholds^11^ 13](#_TOC_250004)

[**Flowchart 12.** Summary of the instrument presented in the article: Too Many Patients. A Framework to Guide Statewide Allocation of Scarce Mechanical Ventilation During Disasters^12^ 14](#_TOC_250003)

[**Flowchart 13.** Summary of the instrument presented in the article: Recomendaciones eticas para la toma de decisiones difıciles en las unidades de cuidados intensivos ante la situacion excepcional de crisis por la pandemia por covid-19: revision rapida y consenso de expertos^13^ 15](#_TOC_250002)

[**Flowchart 14.** Summary of the instrument presented in the article: A Framework for Rationing Ventilators and Critical Care Beds During the COVID-19 Pandemic^14^ 16](#_TOC_250001)

[**Flowchart 15.** Summary of the instrument presented in the article: COVID-19 pandemic: triage for intensive-care treatment under resource scarcity^15^ 17](#_TOC_250000)

## **Flowchart 1.** Summary of the instrument presented in the article: Criteria for prioritizing access to healthcare resources in New Zealand during an influenza pandemic or at other times of overwhelming demand^1^

### NO

**Normal threshold question**: Does the patient meet the clinical criteria for access to the resource during normal times (that is, when there is not overwhelming demand for the resource)?

Patient doesn’t access the resource.

YES

NO

**Competition question:** Are there other patients who meet the normal clinical criteria (as per question 1), who are competing for the same resource (which is currently insufficient to accommodate all of the patients who are competing)?

Patient accesses the resource

go to question 2.

### YES

go to question 3.

### YES

**Alternative options question:** Can any of the competing patients (including those who are already using the resource) have alternative care which, although perhaps not the first choice, will provide reasonably similar benefit to the patient and not cause significant harm due to accessing the alternative rather than the original choice?

Appropriate patient(s) access the alternative care

NO for any remaining patients, go to question 4.

### YES

**Deferability question:** Can any of the competing patients have their access to the resource deferred to a future time when demand is likely to be less, without coming to significant harm

Defer access

NO or any remaining patients, go to question 5.

f

### YES

**Expansion question:** Can the resource be expanded to accommodate greater access, perhaps by redistribution of resources from services which are not experiencing overwhelming demand, or from services which can be deferred without significant harm to patients?

Expand resource

NO for any remaining patients, go to question 6.

### YES

Yes – consider how effective the mitigating options will be and go to question 7.

**Mitigation question:** After consideration of questions 1 to 5, there are still more patients needing to access the resource than the resource can accommodate.

Are there any alternative options for any of the competing patients, which will mitigate the harms of missing out on the resource in question?

NO go to question 7.

### YES

**Ranking question**: After consideration of questions 1 to 5, there are still more patients needing to access the resource than the resource can accommodate. Of those competing for the resource, (including those who are already using the resource) ‘rank’ them in order of perceived ‘net benefit’ of accessing the resource – that is, the sum of the estimated benefit of access to the resource and the harm of not accessing the resource. The ‘net benefit’ should also take into account any mitigation of harm arising from the options identified in question 6.

Can patients be ranked to clearly differentiate their net benefit of accessing the resource?

Those whose ‘net benefit’ ranks higher should access the resource before those whose ‘net benefit’ ranks lower.

NO go to question 8.

**Final question**: If the competing patients cannot be differentiated in terms of ‘net benefit’ then fairness suggests they should access the resource according to who sought access first. If they cannot be differentiated on a ‘first come, first served’ basis then access can be determined by a process of equal and unbiased chance such as tossing a coin or use of a ballot.

## **Flowchart 2**. Summary of the instrument presented in the article: Development of a triage protocol for critical care during an influenza pandemic^2^

| **Triage Code** | **Criteria** | **Action or priority** |
| --- | --- | --- |
| Blue | Exclusion criteria met or SOFA score >11 | Manage medically, Provide palliative care as needed  Discharge from critical care |
| Red | SOFA score < or = 7 or single-organ failure | Highest priority |
| Yellow | SOFA score 8-11 | Intermediate priority |
| Green | No significant organ failure | Defer or discharge Reassess as needed |


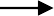


Does the patient meet the inclusion criteria?

- **Requirement for invasive ventilatory support:** refractory hypoxemia (SpO2 <90% or non-rebreather mask or FiO2 > 0,85; respiratory acidosis (pH <7,2), clinical evidence of impending respiratory failure, inability to protect or maintain airway.
- **Hypotension** (systolic blood pressure <90 mmHg or relative hypotension) with clinical evidence of shock (altered level of consciousness, decreased urine output or order evident of end-organ failure) refractory to volume resuscitation requiring vasopressor or inotrope support that cannot be manage in ward setting.

### YES


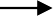


Does the patient have one of the exclusion criteria?

* also apply the SOFA

The patient is excluded from admission or transfer to critical care if any of the following is presented:

**Exclusion criteria**: severe trauma; severe burns of patient with any 2 of the following: Age >60, 40% of total body surface area affected, inhalation injury; cardiac arrest: unwitnessed arrest, not responsive to electrical therapy (defibrillation or pacing), recurrent cardiac arrest; severe baseline cognitive impairment; advanced untreatable neuromuscular disease; metastatic malignant disease, advanced and irreversible neurologic or event or condition; Age > 85 years; elective palliative surgery; end-stage organ failure meeting the following criteria:

Heart: NYHA class III or IV heart failure;

Lungs: COPD with FEV < 25% predicted baseline; PaO2 <55 mmHg or secondary pulmonary hypertension; cystic fibrosis with postbronchodilator FEV < 30% or baseline PaO2 <55 mmHg; pulmonary fibrosis with VC or TLC < 60% predicted, baseline PaO2

<55 mmHg or secondary pulmonary hypertension; primary pulmonary hypertension with NYHA class III or IV heart failure, right atrial pressure > 10 mmHg, or mean pulmonary arterial pressure > 50 mmHg;

Liver: Child-Pugh score > or = 7.

NO


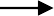


Admit to the ICU

YES

Daily reassessment: evaluate the development of exclusion criteria after 48h and 120h

## **Flowchart 3.** Summary of the instrument presented in the article: Concept of Operations for Triage of Mechanical Ventilation in an Epidemic^3^

Tier 1: Do not offer AND withdraw ventilatory support for patients with any one of the following:

Tier 2: Do not offer AND withdraw ventilatory support from patients with respiratory failure requiring intubation with the following

conditions (in addition to those in tier 1):

|  | **DEVELOPMENT OF CRITERIA FOR RESTRICTION OF MECHANICAL** | |  |
| --- | --- | --- | --- |
|  | |  | |
| Criteria should be implemented in a tiered or stepwise fashion, so that as resources are exhausted, another (stricter) tier of exclusion criteria is implemented in an attempt to provide the best care possible to those with the best chance of survival. | | | |

Tier 3: Specific protocols to be agreed upon by guideline development committee. Possibilities include:

1. Respiratory failure requiring intubation with persistent hypotension (systolic blood pressure < 90 mmHg for adults) unresponsive to adequate fluid resuscitation after 6–12 hours of therapy and signs of additional end-organ dysfunction (e.g., oliguria, mental status changes, cardiac ischemia)
2. Failure to respond to mechanical ventilation (no improvement in oxygenation or lung compliance) and antibiotics after 72 hours of treatment for a bacterial pathogen (timeline may be modified based on organism-specific data)
3. Laboratory or clinical evidence of R4 organ systems failing
   1. Pulmonary (adult respiratory distress syndrome, ventilatory failure, refractory hypoxemia)
   2. Cardiovascular (left ventricular dysfunction, hypotension, new ischemia)
   3. Renal (hyperkalemia, diminished urine output despite adequate fluid resuscitation, increasing creatinine level)
   4. Hepatic (transaminase greater than two times normal upper limit, increasing bilirubin or ammonia levels)
   5. Neurologic (altered mental status not related to volume status, metabolic, or hypoxic source, stroke)
   6. Hematologic (clinical or laboratory evidence of disseminated intravascular coagulation)

Patients with pre-existing system compromise or failure including:

1. Known congestive heart failure with ejection fraction < 25% (or persistent ischemia unresponsive to therapy and pulmonary edema).
2. Acute renal failure requiring hemodialysis (related to illness).
3. Severe chronic lung disease including pulmonary fibrosis, cystic fibrosis, obstructive or restrictive diseases requiring continuous home oxygen use before onset of acute illness.
4. Acquired immunodeficiency syndrome (AIDS), other immunodeficiency syndromes at stage of disease susceptible to opportunistic pathogens (e.g., CD4 <200 for AIDS) with respiratory failure requiring intubation.
5. Active malignancy with poor potential for survival (e.g., metastatic malignancy, pancreatic cancer)
6. Cirrhosis with ascites, history of variceal bleeding, fixed coagulopathy, or encephalopathy.
7. Acute hepatic failure with hyperammonemia
8. Irreversible neurologic impairment that makes patient dependent for personal cares (e.g., severe stroke, congenital syndrome, persistent vegetative state)
9. Restriction of treatment based on disease-specific epidemiology and survival data for patient subgroups (may include age-based criteria)
10. Expansion of preexisting disease classes that will not be offered ventilatory support.
11. Applying Sequential Organ Failure Assessment scoring to the triage process and establishing a cutoff score above which mechanical ventilation will not be offered.

## **Flowchart 4.** Summary of the instrument presented in the article: Definitive Care for the Critically Ill During a Disaster: A Framework for Allocation of Scarce Resources in Mass Critical Care^4^

**1° Patient Assessment**

**Inclusion Criteria:** patients in need of intensive care, who need to use a mechanical ventilator, vasopressors or who have another active need for intensive care.

**Exclusion Criteria:** mortality ≥ 80%; SOFA > 15; SOFA > 5 for ≥ 5 days and with flat or rising trend; ≥ 6 organ failures; severe chronic disease with a short life expectancy; severe trauma; severe burns on patient with any two of the following: age > 60 yr, > 40% of total body surface area affected, inhalational injury; unwitnessed cardiac arrest or witnessed cardiac arrest not responsive to electrical therapy (defibrillation or pacing) or recurrent cardiac arrest; severe baseline cognitive impairment; advanced untreatable neuromuscular disease; metastatic malignant disease; advanced and irreversible neurologic event or condition; New York Heart Association class III or IV heart failure; COPD with FEV1 < 25% predicted, baseline Pao2 <55 mm Hg or secondary pulmonary hypertension; cystic fibrosis with postbronchodilator FEV1 < 30% or baseline Pao2 < 55 mm Hg; pulmonary fibrosis with vital capacity or total lung capacity < 60% of predicted, baseline Pao2 < 55 mm Hg, or secondary pulmonary hypertension; primary pulmonary hypertension with New York Heart Association class III or IV heart failure, right atrial pressure > 10 mm Hg, or mean pulmonary arterial pressure > 50 mm Hg; Child-Pugh score ≥ 7; age > 85 yr or elective palliative surgery.

**The Triage Process**

**2° Patient classification:** using the SOFA scale

**3º Resource allocation:** definition of patients eligible or not for intensive care and use of a mechanical ventilator.

Based on the inclusion and exclusion criteria as well as on the SOFA score and tiebreaker criteria, the triage officer (usually a highly experienced surgeon) and the team will determine whether there is sufficient basis to justify the reallocation of resources.


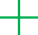


The triage officer is in charge, assesses all patients, assigns a level of priority for each, and directs attention to the highest-priority patients. The triage officer may also be in charge of logistics, such as patient transfers or availability of resources, but is often assigned an administrator/director for this purpose.

## **Flowchart 5.** Summary of the instrument presented in the article: Allocation of Ventilators in a Public Health Disaster^5^

NO


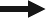


Cardiac Arrest: unwitnessed arrest, recurrent arrest, arrest unresponsive to standard measures, trauma-related arrest.

Metastatic malignancy with poor prognosis, severe burn: body surface area >40%, severe inhalation injury.

End stage organ failure: cardiac: NYHA class III or IV. Pulmonary: severe chronic disease with FEV< 25%. Hepatic: MELD score>20. Renal: dialysis dependent. Neurological: severe, irreversible neurological event/condition with high expected mortality.

Reassessment: The continued use of the ventilator will be reevaluated at intervals of 48 and 120 hours.

Patients who have showed improvement will continue to use the ventilator until the next assessment, while those who have failed to meet the criteria will lose access to mechanical ventilation. Time tests for ventilator use should reflect the expected duration of treatment for severe pulmonary complications.

Does the patient have one of the exclusion criteria?

* also apply the SOFA

They will not receive ventilator

Patients arriving with clinical evidence of impending lung failure?

They will not receive ventilator

YES

YES

| Color code | Criteria | Priority/Action |
| --- | --- | --- |
| Blue | Exclusion Criteria or SOFA>11 | High probability of mortality; should be discharged from critical care; medical management  + or - palliative and d/c |
| Red | SOFA score < or = 7 or single organ failure | Highest priority for critical care |
| Yellow | SOFA score 8-  11 | Intermediate priority for critical care |
| Green | No significant organ failure | Low probability of mortality; defer or d/c, reassess as needed |

NO


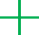


Patients using ventilators in chronic care facilities would not be subjected to acute care triage guidelines. If, however, such patients required transfer to an acute care facility, they would be assessed by the same criteria as all of the other patients and may lose access to continued ventilator use. Chronically ill patients are especially vulnerable in public health emergencies.

## **Flowchart 6.** Summary of the instrument presented in the article: Rationing of resources: ethical issues in disasters and epidemic situations^6^

A color-coded tool for intensive care.

**Inclusion Criteria:** Any patient with ventilatory failure

requiring ventilatory support, regardless of the etiology.

**Firm exclusion criteria**: These are patients presenting with severe trauma, such as severe burns quantified as >40% body surface area and/or severe inhalation injury, cardiac arrest that either is unwitnessed or witnessed without any response or recurrent need for resuscitation; a calculated sequential organ failure assessment (SOFA) >11 (to be discussed), or failure of more than four organ systems.

Cardiac: NYHA class III or IV; Renal: dialysis dependent; Neurological: poor prognosis; Immunocompromised state, i.e., advanced HIV/AIDS; Pulmonary: severe CLD FEN<25%; Hepatic: MELD score >20; Malignancy: poor prognosis

The patient is excluded from admission or transfer to intensive care if any of the following situations are present:

**Relative exclusion criteria:** defined by organ function measures or associated disease prognosis. NOTE: age is not a criterion.

| **4. Checklist of clinical progress**  An intensive care assessment checklist.  Document placed in front of the patient's chart to facilitate patient monitoring.  . | **5. Palliative care protocol**  When the  application of mechanical ventilation becomes medically futile, the  obligation to apply its use is replaced by an obligation to provide comfort and dignity to those dying. | **6. Appeals process**  There may be some conflicts or unforeseen issues that need  arbitration by the Pandemic Triage Committee. | **7. Early family involvement**  Involve the family in the clinical decision-making model from the beginning. The components of the decision model should be made available to family members in a written document or soon after  presentation or admission.  Transparency is essential for the ethical ideal of fidelity and trust. |
| --- | --- | --- | --- |

**2. Phased Allocation of Resources**

Review of needs for ventilation or the prevision of the duration of ventilation support.

Cancellation of elective procedures.

**1. Formation of a Pandemic**

**Triage Committee**

When a medical center faces resource allocation decisions it aims to be neutral, impartial and act as a supervisory body, regularly reviewing current advances and decisions in state, federal and international organizations.

**PRESENTATION OF THE GENERAL ASPECTS OF THE INSTRUMENT**

**3. Clinical Evaluation**

| **SCREENING TOOL FOR CRITICAL CARE** | | |
| --- | --- | --- |
| **Initial Evaluation** | | |
| **Color Code** | **Criteria** | **Priority/Action** |
| Blue | Exclusion Criteria OR SOFA>11 | Medical Management ±Palliative care and D/C |
| Red | SOFA <7 OR Single Organ Failure | Highest |
| Yellow | SOFA 8-11 | Intermediate |
| Green | No significant organ failure | Defer or D/C, re-assess as needed |
|  | | |
| **48-Hour Assessment** | | |
| **Color Code** | **Criteria** | **Priority/Action** |
| Blue | Exclusion Criteria OR SOFA>11 OR SOFA 8-11 and  no change | Medical Management and Palliative and Discharge from intensive care unit |
| Red | SOFA <11 and decreasing | Highest |
| Yellow | SOFA <8 and no change | Intermediate |
| Green | No longer ventilator dependent | Discharge from intensive care unit |
|  | | |
| **120-Hour Assessment** | | |
| **Color Code** | **Criteria** | **Priority/Action** |
| Blue | Exclusion Criteria OR SOFA>11 OR SOFA 8-11 and  no change | Palliative care and Discharge from intensive care unit |
| Red | SOFA <11 and decreasing progressively | Highest |
| Yellow | SOFA <8 and no change (<3 point decrease in past 72h) | Intermediate |
| Green | No longer ventilator dependent | Discharge from intensive care unit |

## **Flowchart 7.** Summary of the instrument presented in the article: Development of a Critical Care Triage Protocol for Pandemic Influenza: Integrating Ethics, Evidence and Effectiveness^7^

1° Patient evaluation: identification of patients who can benefit from admission to intensive care.

**Inclusion criteria**: requirement for invasive ventilatory support (refractory hypoxemia - SpO2

<90% wearing a mask; respiratory acidosis with pH <7.2; clinical evidence of imminent respiratory failure; inability to protect or maintain airways). Hypotension (systolic pressure <90 or relative hypotension with clinical evidence of shock - altered level of consciousness, urine output or other organ failure - refractory to volume resuscitation that requires vasopressor / inotropic support that cannot be managed in the infirmary.

**Exclusion criteria**: severe trauma (ISS score> 16); severe burns if scored on two of the following items: age> 60 years; TBSA> 40% or inhalation injury; unattended cardiac arrest or cardiac arrest that does not respond to electrical therapy (defibrillation, cardioversion or stimulation) or recurrent cardiac arrest; severe cognitive impairment; advanced intractable neuromuscular disease; incurable metastatic malignancy; advanced and irreversible immunocompromise (example: HIV with CD4 <200); severe and irreversible neurological event / condition; heart failure NYHA class III or IV or history of recurrent CHF; COPD with FEV1 <25% predicted, baseline PaO2 <55 mmHg or secondary pulmonary hypertension; HR with FEV1 after bronchodilator <30% or baseline PaO2 <55 mmHg; pulmonary fibrosis with CV or CPT <60% of predicted, baseline PaO2

<55 or secondary pulmonary hypertension; primary pulmonary hypertension with NYHA class III or IV heart failure, right atrial pressure> 10 mmHg or mean pulmonary pressure> 50 mmHg; patients using home oxygen; patients in line for transplants; patients with Child Pugh score ≥ 7, with a history of refractory ascites or hepatic encephalopathy; dialysis patients; over the age of 85 years; patients with a transfusion requirement of> 6 units of blood components within a 24-hour period; patients with elective palliative surgery; patients with guidelines not to resuscitate or "allow natural death", patients with a history of quadriplegia; history of stroke with great

**SCREENING PROCESS**


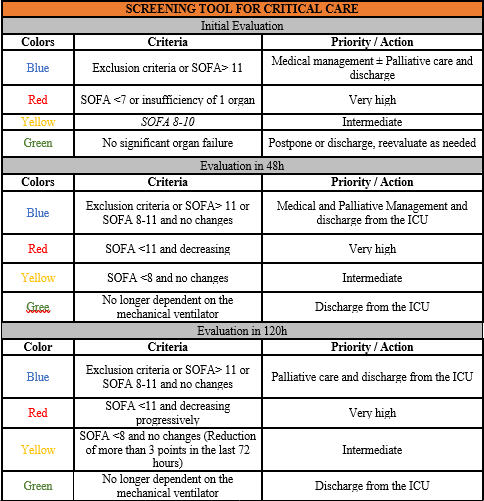


**2° Patient classification** using the SOFA scale and definition of clinical conduct

**Tiebreaker criteria**

Multiplier effect: is the patient an essential services worker?

Exposure in the workplace: is it likely that the patient was exposed during the exercise of his professional duties?

Caregiver: the patient is a pregnant woman, caregiver of children under 18 years old, has adult dependents or elderly people with disabilities?

Prognosis: is there any evidence that this patient is significantly more likely to survive ICU admission and return to a reasonable functional state than other eligible patients who currently require intensive care?

Fair entry or life cycle principle: is the patient in an early stage of life?

## **Flowchart 8.** Summary of the instrument presented in the article: Who Should Receive Life Support During a Public Health Emergency? Using Ethical Principles to Improve Allocation Decisions^8^

**No**

Does the patient need scarce resources?

He/she will not receive treatment

| **RESOURCE ALLOCATION INSTRUMENT** | | | | | |
| --- | --- | --- | --- | --- | --- |
|  | | **Point System** | | | |
| **Principle** | **Specification** | **1** | **2** | **3** | **4** |
| Save the most lives | Prognosis for short  term survival (SOFA score) | SOFA<6 | SOFA 6-9 | SOFA 10-12 | SOFA>12 |
| Save the most years of life | Prognosis for long  term survival (Medical assessment of comorbidities) | No comorbidities which limit long term survival | Minor comorbidities with small impact on long term survival | Major comorbidities with substantial impact on long term  survival | Severe comorbidities; likely death within 1 year |
| Life cycle principle | Prioritize those who have had the least  chance to live through life's stages. (Age  in years) | age 12-40 | age 41-60 | age 61-74 | age > 75 |

**Yes**

He/she will not receive treatment

**No**

When analyzing the points system, the lower the SOFA, the degree of comorbidity, and age, the lower the score. Does the patient have the lowest


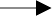
**Yes**

Patient receives treatment

If there is a tie, the ballot criterion is used

**Flowchart 9.** Summary of the instrument presented in the article: Chapter 7. Critical care triage. Recommendations and standard operating procedures for intensive care unit and hospital preparations for an influenza epidemic or mass disaster ^9^

**No**

**Yes**

Daily reassessment and categorization according to the 48 - 120-h criteria for an unlimited period.

Qualified patient for ICU admission.

Patient presents some of the exclusion criteria?

**Requirement for invasive ventilatory support**: Refractory hypoxemia (SpO2

<90% on non-rebreather mask/FiO2 >0.85); Respiratory acidosis with pH <7.2; Clinical evidence of impending respiratory failure; Inability to protect or maintain airway (altered level of consciousness, significant secretions or other airway issue).

**Hypotension:** Hypotension (SBP < 90 mmHg or relative hypotension) with clinical evidence of shock (altered level of consciousness, decreased urine output or other end organ failure) refractory to volume resuscitation requiring vasopressor/inotrope support.

Patient presents some of the inclusion criteria?

**Yes**

| The patient is excluded from admission to critical care if any of the following are present  **Exclusion criteria:** identify patients who are not candidates for ICU admission including patients: (1) with a poor prognosis despite care in an ICU, (2) requiring resources that cannot be provided, (3) whose underlying illness has a poor prognosis with a high likelihood of death and (4) who are “too well”. | | | |
| --- | --- | --- | --- |
|  | **Triage prioritization tool** | | |
|  | **Initial Assessment** | | |
|  | **Collor Code** | **Criteria** | **Priority/Action** |
|  | Blue | Exclusion Criteria or SOFA >11 | Medical Mgmt +/- Palliate & Discharge from critical care |
|  | Red | SOFA ≤ 7 or Single Organ Failure | Highest |
|  | Yellow | SOFA 8 –11 | Intermediate |
|  | Green | No significant organ failure | Defer or Discharge. reassess as needed |
|  |  | | |
|  | **48 hour Assessment** | | |
|  | **Collor Code** | **Criteria** | **Priority/Action** |
|  | Blue | Exclusion Criteria Or SOFA > 11 Or SOFA 8 – 11 | Medical Mgmt +/- Palliate & Discharge from critical care |
|  | Red | SOFA score < 11 and decreasing | Highest |
|  | Yellow | SOFA < 8 | Intermediate |
|  | Green | No longer ventilator dependant | Defer or Discharge, reassess as needed |
|  |  | | |
|  | **120 hour Assessment** | | |
|  | **Collor Code** | **Criteria** | **Priority/Action** |
|  | Blue | Exclusion Criteria or SOFA > 11 or SOFA < 8 | Medical Mgmt +/- Palliate & Discharge from critical care |
|  | Red | SOFA score < 11 and decreasing progressively | Highest |
|  | Yellow | SOFA < 8 with < 3 point decrease in past 72h | Intermediate |
|  | Green | No longer ventilator dependant | Defer or Discharge, reassess as needed |
|  | | | |

## **Flowchart 10.** Summary of the instrument presented in the article: Triage: care of the critically ill and injured during pandemics and disasters: CHEST consensus statement^10^

**DECISION FLOW OF THE SCREENING PROCESS**

**Requirement for invasive ventilatory support**: Refractory hypoxemia (Spo2 <90% on nonrebreather mask Fio2>0.85) Respiratory acidosis with pH<7.2

Clinical

evidence

of

respiratory

failure

Inability to protect or maintain airway.

**Hypotension:** Hypotension refractory to volume resuscitation and need for vasopressor / inotropic support.

Does the patient meet the inclusion criteria?

**No**

Monitor and reassess as needed

**Yes**

The patient is excluded from intensive care if any of the following situations are present:

**Low Probability of Survival Exclusion Criteria:** Cardiac arrest; Severe trauma (Trauma Injury Severity Score with predicted mortality

<90%); Severe burns (Predicted mortality>90%); Severe and irreversible neurologic event or condition; Severe prematurity (<24 wk estimated gestational age).

**Short Life Expectancy Exclusion Criteria:** Metastatic malignancies; Hematologic malignancies with poor prognosis; End-stage organ failure with expected survival <1 year, such as end-stage cardiac failure (NYHA class IV), severe chronic lung disease, advanced hepatic failure (MELD score >20); Very advanced age; Advanced and irreversibly immunocompromised, such as drug-resistant AIDS; Congenital anomalies with expected survival <1 year.

Do the patients meet the exclusion criteria?

**No**

Relative exclusion criteria**:** Admission to the available ICU bed based on


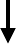


**Yes**

**No**

The attempted treatment is successful (improvement)

Discharge or palliative care

Daily reassessment: evaluate the development of exclusion criteria.

Failure in treatment

Palliative care

After 72 hours of attempt at treatment: has the patient met the aims of the trial and is showing significant evidence of improvement?

**Recovered**

**HOSPITAL DISCHARGE**

## **Flowchart 11.** Summary of the instrument presented in the article: Pediatric Triage in a Severe Pandemic: Maximizing Survival by Establishing Triage Thresholds^11^

Casualty presentes intubated

**Yes**

**EXCLUDE:**

Too sick

**No**

Is Probability of death

>= Threshold?

**Yes**

Is Days on Ventilation

>= threshold?

**EXCLUDE:**

Too sick

**No**

**ADMIT**:

Optimal for treatment

**No**

**Yes**

Is PICU bed available?

**No**

Enter time-limited queue to wait for free PICU bed

Did PICU bed become open in time?

Casualty expires before treatment

Casualty presents non- intubated

**EXCLUDE:**

Too Healthy

## **Flowchart 12.** Summary of the instrument presented in the article: Too Many Patients. A Framework to Guide Statewide Allocation of Scarce Mechanical Ventilation During Disasters^12^

**Yes**

Did the patient have uncontrolled cardiac arrest, is he/she unresponsive to recurrent defibrillation, or advanced and irreversible neurological stimulation/event, or is he/she over 60 years old and had 50% of the body area burned?

Exclude the patient

**No**

| **Proposed Strategy for Ventilator Allocation in Epidemics of Novel Respiratory Pathogens** | | | | | |
| --- | --- | --- | --- | --- | --- |
|  | | Point System | | | |
| Principle | Specification | 11 | 2 | 3 | 4 |
| Prognosis for short-term survival | Adults (SOFA) or  pediatrics (PELOD-2) | SOFA < or  = 8 | SOFA 9-11 | SOFA 12-  14 | SOFA> 14 |
| - | - | PELOD-2  < or = 12 | PELOD-2 12-13 | PELOD-2 14-16 | PELOD-2  > or = 17 |
| Prognosis for long-term survival | Prognosis for long-term survival (assessment of comorbid conditions) |  |  | Severe comorbid conditions; death likely within 1 year |  |

**No**

When analyzing the points system, does the patient have the lowest

Exclude the patient

**Yes**

PATIENT RECEIVES TREATMENT

If there is a tie, the age criterion is used

| **Proposed Strategy for Ventilator Allocation in Epidemics of Novel Respiratory Pathogens** | | | | | |
| --- | --- | --- | --- | --- | --- |
|  | | Point System | | | |
| Principle | Specification | 11 | 2 | 3 | 4 |
| Secondary consideration: Lifecycle | Prioritize those who have had the lease  chance to live through life’s stages (age) | age 0-49 | age 50-  69 | age 70-  84 | age > 85 |


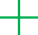


If a pregnant patient presents in respiratory failure requiring mechanical ventilation, obstetrical evaluation of fetal heart tones should be performed urgently. Those individuals with a healthy fetus based on this evaluation will be given a 1-point “credit” (reduction) on their priority score, thus giving them higher priority.

## **Flowchart 13.** Summary of the instrument presented in the article: Recomendaciones eticas para la toma de decisiones difıciles en las unidades de cuidados intensivos ante la situacion excepcional de crisis por la pandemia por covid-19: revision rapida y consenso de expertos^13^

**General recommendations**

Organization

Characteristics and global situation of the person

3. Better allocation of resources, proposing to adopt a model.

**Priority 2:**

Needs monitoring and may need immediate intervention (at the moment without mechanical ventilation).

**Priority 1:**

Critical and unstable, they need intensive monitoring and treatment.

Age> 80 years with or without comorbidity - high concentration oxygen mask, high flow oxygen therapy or non-invasive ventilation (last 2 consider the risk / benefit ratio for the production of aerosols. IMV will be carefully selected depending on the risks / benefits in patient without comorbidities.

70-80 years without major comorbidity- can use IMV.

70-80 years with moderate / severe comorbidity - do not use IMV

Dementia or neurodegenerative disease - do not use IMV

In other cases - remove IMV according to the daily SOFA score

* IMV- invasive mechanical ventilation

1. Apply specific ethical criteria to situations, defending an allocation strategy that maximizes survival of hospital discharge (chronological age should not be the only element in allocation strategies).
2. The screening is based on the principles of distributive justice, prioritizing the best “cost / opportunity” and proportionality.
3. Recommends carrying out the decision-making process between the team, the patient and the family.
4. Need for a territorial contingency plan.
5. Optimization of human, structural and material resources.
6. Consensus of common technical and ethical criteria.
7. Plan alternatives, mainly to people undergoing palliative treatment.
8. Starting from the need to carry out a comprehensive assessment in a global situation or “biological age” and its values and preferences - including the possibility of having an advanced directive document or an advanced care plan.
9. Evaluation of palliative care with quality.

Ethics of decision

| **Priority 3:**  Unstable and critical, presence of comorbidity, with little chance of recovery. | |  | **Priority 4:**  Minimum benefit or irreversible terminal illness. | |
| --- | --- | --- | --- | --- |
|  |  | | |  |

**Ventilation mode depending on the person's characteristics**

**Classifies candidates with acute respiratory failure to enter the ICU**


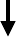


Forward to ICU

Intermediate care

Do not refer to ICU

## **Flowchart 14.** Summary of the instrument presented in the article: A Framework for Rationing Ventilators and Critical Care Beds During the COVID-19 Pandemic^14^

**PRESENTATION OF THE INSTRUMENT GENERAL ASPECTS**

**2. ICU / Ventilators** Allocation Process Guidelines: the scoring system applies to all critical patients. Ethical objective of the allocation structure: Consistent with accepted standards during public health emergencies - the main

objective of the allocation structure is to offer maximum benefit to the greatest number of people.

**1. Formation of the Triage Team** Triage officer: Desirable qualities for triage officers include being a physician with proven experience in treating critical patients, strong leadership, communication and conflict resolution skills.

They should aim to benefit the greatest number of patients. Empowered to make decisions related to the reallocation of intensive care resources previously allocated, without personal principles and beliefs.

Triage team: formed by a nurse (with experience in the ICU) and administrative assistant. It should support and assist in the official's decisions and document the process, liaising with the hospital's senior management. A hospital management representative must be on the team to oversee team maintenance.

| **4. Step 2**  Applying the Multiple Principles Strategy  Determine scores daily, even twice a day.  Revaluation whenever necessary.  Option for use:  Color scale or number score:  Red - high priority (score 1-3)  Orange -  intermediate priority (score 4-5)  Yellow - low priority (score 6-8). | | **5. Resolving ties**  -life-cycle considerations should be used as the first tiebreaker, with priority  going to younger patients.   - We also   recommend that individuals who are vital to the acute care response be given priority, which could be operationalized in the form of a tiebreaker. Exclude front-line staff.   - If there are still ties after these two tiebreakers are applied, a lottery (i.e., random allocation) should be used to break the tie. | **6. Reassessment**  Decrease the chance of arbitrary decisions. The Triage Team will carry out periodic reassessments.  A multidimensional assessment should be used to quantify changes in patients. Patients who show improvement will  continue with intensive care / ventilation until the next assessment. If there are patients waiting in line, beds are scarce and the patient is not  responding to treatment, there will be a relocation of patients. After the disclosure of this decision, the patient and / or family must be communicated.  Patients who experience a severe acute event or a highly morbid complication, the Triage Team can make the decision that the patient is no longer eligible for treatment. |
| --- | --- | --- | --- |
| **ublic Health Eme**  INTS SYSTEM | **rgency** |  |  |
| **3** | **4** | |  |
| 8 SOFA 9-11 | SOFA ≥12 | |  |
| d s  al ---  n m | Severely life-limiting conditions; death likely within 1 year | |  |

**3. Step 1**

| **Multi-principle Strategy to Allocate Critical Care/Ventilators During a P** | | | | | |
| --- | --- | --- | --- | --- | --- |
|  | | PO | | | |
| Principle | **Specification** | **1** | **2** |  |  |
| Save the most lives | Prognosis for short-term survival (SOFA  score) | SOFA <6 | SOFA 6- |  |  |
| Save the most life-years | Prognosis for long-term survival (medical  assessment of comorbid conditions) | --- | Major comorbi condition with substanti impact o  long-ter survival |  |  |

##
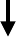

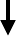

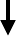

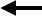
**Flowchart 15.** Summary of the instrument presented in the article: COVID-19 pandemic: triage for intensive-care treatment under resource scarcity^15^


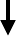

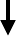

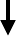

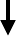

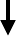

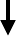

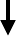


**Step 2:** Presence of both of the following criteria:

- Stabilisation or improvement of oxygenation and ventilation, or of the underlying organ dysfunction
- Stabilisation

improvement hemodynamics.

or of


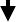


**Step 2 – Exclusion Criteria**

Stage B (**)

- Severe trauma

- Severe burns (>40% of total body surface area affected) with inhalation injury
- Severe cerebral deficits after stroke
- Chronic condition:
- NYHA class III or IV heart failure
- COPD GOLD 4 (D) or COPD A–D with either FEV1 <25% or cor pulmonale or home oxygen therapy (longterm oxygen therapy)
- Liver cirrhosis with refractory ascites or encephalopathy > stage I
- Stage V chronic kidney disease (KDIGO)
- Moderate dementia (confirmed)
- Age >85 years

–Age >75 years and at least one criterion: (Liver cirrhosis; Stage III chronic kidney disease (KDIGO); NYHA class >I heart failure; Estimated survival <24 months).

Stage A (*)

- Patient’s wishes (advance directive, etc.)
- Unwitnessed cardiac arrest, recurrent cardiac arrest, cardiac arrest with no return of spontaneous circulation
- Malignant disease with a life expectancy of less than 12 months
- End-stage neurodegenerative disease
- Severe and irreversible neurological event or condition
- Chronic condition:
- NYHA class IV heart failure
- COPD GOLD 4 (D)
- Liver cirrhosis, Child-Pugh score >8
- Severe dementia
- Severe circulatory failure, treatment-resistant despite increased vasoactive dose (hypotension and/or persistent inadequate organ perfusion)
- Estimated survival <12 months


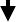


**If one of the exclusion criteria is fulfilled, the patient is not to be admitted to the ICU**

The presence of one criterion means that continuation of ICU treatment is no longer indicated and the patient will receive palliative care

Stage A (*)

– Occurrence of cardiac arrest during ICU stay, unless resuscitation

with defibrillation is successful;

- Persistence or development of a significant triple organ failure.

Stage B (**)

- No improvement in respiratory or hemodynamic status, or in the underlying organ dysfunction;
- occurrence of cardiac arrest during ICU stay;
- persistence or development of a significant dual organ failure.

**Step 3:** Presence of one of the following criteria for little or no likelihood of benefit with ICU treatment:

**Step 1:** Presence of a criterion for ICU discharge:

– Patient extubated or with spontaneous breathing (or partial ventilatory support) through tracheotomy → **Patient discharged from ICU**

**Short-term prognosis is decisive** for purposes of triage. For ICU admission, highest priority is to be accorded to those patients whose prognosis with regard to hospital discharge is good with intensive care, but poor without it – i.e. the patients who will benefit most from intensive care.

**Age** in itself is not to be applied as a criterion, however, it´s indirectly taken into account under the main criterion “short-term prognosis”, since older people more frequently suffer from comorbidity. In connection with COVID-19, age is a risk factor for mortality and must therefore be taken into account.

1. **Criteria for ICU triage**

| **- On admission:** extent and duration of treatment, determination  of pathway (e.g. ICU treatment, intermediate  care, palliative care). | To make decisions in such a way as to save the largest possible number of lives.  **Stage A (*)**: *ICU beds available, but capacity limited* |
| --- | --- |
| – **After 2–3 days:** continuation of treatment, limitation of treatment intensity or duration, modification of treatment goal and palliative care. | **Stage B** (**): ***No ICU beds available;***  cardiopulmonary resuscitation is not to be undertaken, except for very brief resuscitation measures in the event of a cardiac arrest occurring in the course of medical interventions (e.g. asystole during spinal anaesthesia). |

**PRESENTATION OF THE INSTRUMENT GENERAL ASPECTS**

| 2. **Triage decisions** | 3. **Decision-making processes** |
| --- | --- |
|  | - The decision-making process must be managed by experienced professionals. Whenever possible, decisions must be made within an interprofessional team. - ICU must be able to make rapid, independent decisions at any time on patient admissions and transfers. - Confidence must be maintained under the most difficult conditions. For this reason, fair rationing criteria and fair processes must be transparently applied at all times. Clear reasons for according (or failing to accord) priority must be documented and updated. Individual decisions must be amenable to examination (they must de documented). Any deviation from the specified criteria must be similarly documented. In addition, mechanisms should be in place for subsequent review of conflicts. |

**2.1 Phases**

**2.2 Stages - short-term prognosis criteria**

**2.3 *Initial triage: criteria for ICU admission***

**2.4 *Triage during ICU stay***

**Step 1 – Inclusion Criteria**: Requirement for invasive ventilatory support? Requirement for hemodynamic support with

**Yes**

**References**

1. Ardagh M. Criteria for prioritizing access to healthcare resources in New Zealand during an influenza pandemic or at other times of overwhelming demand. N Z Med J 2006; 119(1243): U2256.
2. Christian MD, Hawryluck L, Wax RS, et al. Development of a triage protocol for critical care during an influenza pandemic. CMAJ 2006;175(11):1377–81.
3. Hick JL, O’Laughlin DT. Concept of operations for triage of mechanical ventilation in an epidemic. Acad Emerg Med 2006;13(2):223–9.
4. Devereaux AV, Dichter JR, Christian MD, et al. Definitive care for the critically ill during a disaster: a framework for allocation of scarce resources in mass critical care: from a Task Force for Mass Critical Care summit meeting, January 26-27, 2007, Chicago, IL. Chest 2008;133(5 Suppl):51S-66S.
5. Powell T, Christ KC, Birkhead GS. Allocation of ventilators in a public health disaster. Disaster Med Public Health Prep 2008; 2(1):20–6.
6. Lin JY, Anderson-Shaw L. Rationing of resources: ethical issues in disasters and epidemic situations. Prehosp Disaster Med 2009;24(3):215–21.
7. Frolic A, Kata A, Kraus P. Development of a critical care triage protocol for pandemic influenza: integrating ethics, evidence and effectiveness. Healthc Q 2009;12(4):54–62.
8. White DB, Katz MH, Luce JM, Lo B. Who should receive life support during a public health emergency? Using ethical principles to improve allocation decisions. Ann Intern Med 2009;150(2):132–8.
9. Christian MD, Joynt GM, Hick JL, Colvin J, Danis M, Sprung CL. Chapter 7. Critical care triage. Recommendations and standard operating procedures for intensive care unit and hospital preparations for an influenza epidemic or mass disaster. Intensive Care Med 2010;36 Suppl 1(Suppl 1): S55-64.
10. Christian MD, Sprung CL, King MA, et al. Triage: care of the critically ill and injured during pandemics and disasters: CHEST consensus statement. Chest 2014;146(4 Suppl): e61S-74S.
11. Gall C, Wetzel R, Kolker A, Kanter RK, Toltzis P. Pediatric Triage in a Severe Pandemic: Maximizing Survival by Establishing Triage Thresholds. Crit Care Med 2016;44(9):1762–8.
12. Daugherty Biddison EL, Faden R, Gwon HS, et al. Too Many Patients…A Framework to Guide Statewide Allocation of Scarce Mechanical Ventilation During Disasters. Chest 2019;155(4):848–54.
13. Rubio O, Estella A, Cabre L, et al. Recomendaciones éticas para la toma de decisiones difíciles en las unidades de cuidados intensivos ante la situación excepcional de crisis por la pandemia por covid-19: revisión rápida y consenso de expertos. Med Intensiva 2020 Apr 15 (Epub ahead of print).
14. White DB, Lo B. A Framework for Rationing Ventilators and Critical Care Beds During the COVID-19 Pandemic. JAMA 2020 Mar 27 (Epub ahead of print).
15. Swiss Academy of Medical Sciences. COVID-19 pandemic: triage for intensive-care treatment under resource scarcity. Swiss Med Wkly 2020; 150:w20229.
